# A Proposed Approach for Joint Modeling of the Longitudinal and Time-To-Event Data in Heterogeneous Populations: An Application to HIV/AIDS's Disease

**DOI:** 10.1155/2018/7409284

**Published:** 2018-01-09

**Authors:** Narges Roustaei, Seyyed Mohammad Taghi Ayatollahi, Najaf Zare

**Affiliations:** Department of Biostatistics, School of Medicine, Shiraz University of Medical Sciences, Shiraz, Iran

## Abstract

In recent years, the joint models have been widely used for modeling the longitudinal and time-to-event data simultaneously. In this study, we proposed an approach (PA) to study the longitudinal and survival outcomes simultaneously in heterogeneous populations. PA relaxes the assumption of conditional independence (CI). We also compared PA with joint latent class model (JLCM) and separate approach (SA) for various sample sizes (150, 300, and 600) and different association parameters (0, 0.2, and 0.5). The average bias of parameters estimation (AB-PE), average SE of parameters estimation (ASE-PE), and coverage probability of the 95% confidence interval (CP) among the three approaches were compared. In most cases, when the sample sizes increased, AB-PE and ASE-PE decreased for the three approaches, and CP got closer to the nominal level of 0.95. When there was a considerable association, PA in comparison with SA and JLCM performed better in the sense that PA had the smallest AB-PE and ASE-PE for the longitudinal submodel among the three approaches for the small and moderate sample sizes. Moreover, JLCM was desirable for the none-association and the large sample size. Finally, the evaluated approaches were applied on a real HIV/AIDS dataset for validation, and the results were compared.

## 1. Introduction

In many studies, the repeated measures of a biomarker are recorded together with time to an event of interest. For example, in HIV/AIDS studies, the trajectories of CD4 counts and time-to-death are collected. In such studies, the interest often lies in understanding the relationships between the longitudinal history of a process and its effect on the risk of an event [1–9].

Classical models such as the separate analysis were performed for these types of data; consequently, the association between the longitudinal and survival outcomes is neglected because the linear mixed model for repeated measurements and the Cox model for time-to-event are conducted separately [6, 10, 11]. In addition, some practices consider the dependency between the two outcomes. Hence, the extended Cox model is used to incorporate the repeated measures as time-varying covariates [4]. In this method, time varying covariates are assumed to be observed continuously till the study terminated using this approach. In practice, this assumption usually does not stratify. Moreover, longitudinal biomarkers tend to be measured with error; thus, modeling the longitudinal measures by a mixed model accounts for this measurement error, which is neglected in the extended Cox model, thus leading to biased and inefficient estimates [4, 10, 12–14].

In recent years, joint model has been used to analyze the longitudinal and survival outcomes simultaneously to consider association between the two outcomes [1, 14, 15]. Joint model enjoys some advantages as compared to classical approaches such as Cox and linear mixed models alone and provides more powerful, accurate, efficient, and robust estimations [4, 10, 12, 16].

Most of the joint models allow subjects to just follow one pattern [5, 6, 13], and the baseline hazard is considered the same for all subjects. Thus, they become inappropriate when there are subgroups with different patterns of response profiles [13].

Joint latent class model (JLCM) is a type of joint models that assumes the population of the subjects to be heterogeneous with multiple homogenous patterns; it is known as the latent class (subpopulation, subtype, or subgroup), having its own longitudinal trajectory and survival curve [2, 5, 6, 17].

Conditional independence (CI) as a fundamental assumption of the JLCM shows that the entire association between longitudinal and survival outcomes is captured by the latent class structure. Thus, given these latent classes, the two types of outcomes are independent [17–20]. However, the CI assumption may not sufficiently show the strength of association and might underestimate the association between the longitudinal and survival processes [13]. Furthermore, to ensure the CI assumption, JLCM has to be examined for various numbers of latent classes, which may ultimately lead to choosing an inappropriate and meaningless size of classes.

We designed a simulation study to combine the joint model with the latent class framework which proposed an approach (PA) for heterogeneous population of subjects free from the CI assumption. At first, the class membership for each subject based on the latent class framework was identified for appropriate number of latent classes. Then, the joint model for longitudinal and survival processes was conducted separately in each latent class for PA. In addition, the separate approach (SA), the linear mixed model for the longitudinal data, and the extended Cox model for the survival outcome were applied separately in each latent class. Finally, we compared PA with JLCM and SA for various sample sizes and different association parameters. In addition, we focused on both the longitudinal and survival outcomes in this study.

## 2. Materials and Methods

### 2.1. Models Framework

#### 2.1.1. Joint Latent Class Model (JLCM)

JLCM assumes that the subjects in each latent class have their own specific longitudinal trajectory and risk of the event, which is useful in many types of research with different patterns of the longitudinal and survival outcomes. In addition, JLCM can be performed for normal and nonnormal distributions and ordinal outcomes [6, 21]. This model does not require normal distribution of random-effects assumption, since it consists of several subpopulations, where this assumption is not realistic [22].

JLCM includes three components: the latent class membership, the longitudinal, and survival submodels. Given the latent class *g*, there is no association between two processes of the longitudinal and survival outcomes; consequently, dependency between time-to-event and longitudinal processes is captured by the structure of latent class [5]. Several methods were introduced to evaluate the CI assumption: evaluation based on the posterior classification, analysis of the residuals conditional on the event, and a score test [19, 23, 24]. Among these approaches, the score test is more powerful than the other methods to assess the CI assumption [2, 5].

In practice, JLCM is applied to a number of latent classes from one to three; the appropriate number of latent classes is determined using the best Bayesian information criterion (lower BIC) and satisfactory CI assumption [6, 20].

Each subject is assigned to each latent class, which has the highest class membership probabilities [25]. A case that is wrongly classified is called misclassified on a categorical variable [13].

#### 2.1.2. Separate Approach (SA)

Commonly, the linear mixed model is used for continuous longitudinal measurements. Also, the parametric or semiparametric survival models are used for modeling the time-to-event data [11]. In SA, the probability that a subject belongs to a latent class structure can be modeled via a latent class framework. Next, the linear mixed model for modeling the longitudinal measurements and the extended Cox model by incorporating repeated measurements into the survival data were conducted for each latent class.

#### 2.1.3. Proposed Approach (PA)

We incorporated the latent class framework to identify its subgroups behind the observed longitudinal measurements and survival outcome. PA provides an approach that achieves appropriate number of the latent classes in heterogeneous populations without requiring the CI assumption. Appropriate number of latent classes are determined by a suitable and easier interpretation according to researcher' comments. For PA, each subject was allocated to an appropriate class according to the highest class membership probabilities. Then, joint model was conducted for each class; additionally, in each latent class, the association between the longitudinal and time-to-event data was modeled by the entire longitudinal trajectory as a covariate in the survival submodel. 

(*1) Latent Class Framework*. The class membership probability for a subject belonging to a latent class can be modeled via a multinomial logistic regression with vector of covariate *X*_*pi*_:

(1) $$\begin{array}{c} \pi_{ig}=P\left(\;c_{i}=g\;|\;X_{pi}\;\right)=\frac{\exp\left(\eta_{0g}+X_{pi}^{T}\eta_{1g}\right)}{\sum_{l=1}^{G}\exp\left(\eta_{0l}+X_{pi}^{T}\eta_{1l}\right)}. \end{array}$$

Let *c*_*i*_ represent the latent variable with *g* = 1,…, *G* latent classes.

*η*_0*g*_ is the intercept for class *g* and *η*_1*g*_ is the vector of class-specific parameters associated with the set covariates *X*_*pi*_. Also, to ensure identifiability, *η*_0*G*_ = 0 and *η*_1*G*_ = 0, that is, last latent class as [2, 5, 25].

In the application, parameters from latent class framework are estimated by maximizing the log likelihood function with iteration of Expected-Maximization (EM) algorithm with steps of Newton-Raphson [26, 27]. 

(*2) Longitudinal Submodel*. The longitudinal submodel is specified as a class-specific linear mixed model. Let *N* be the total number of subjects and let *j* = 1,2,…, *n*_*i*_ be the number of repeated measurements for subject *i*. The longitudinal submodel given to each latent class can be written as

(2) Yitijci=g=Zitijbig+Xitijβg+ϵitij=Zig∗tij+ϵitij.

Given the latent class *g*, *Y*_*i*_(*t*_*ij*_) is the longitudinal outcome for subject *i* at the time of *t*_*ij*_, and *Z*_*i*_(*t*_*ij*_) represents the random effect covariate vectors at the time *t*_*ij*_ for subject *i*, associated with the *p*-vector of random effect *b*_*ig*_, where *X*_*i*_(*t*_*ij*_) is the fixed effects covariate vectors at the time *t*_*ij*_, which is associated with the *q*-vector of fixed effect. The random error term, *ϵ*_*i*_(*t*_*ij*_) is usually assumed to be normally distributed. 

(*3) Survival Submodel*. The survival submodel is specified as a Cox or any parametric survival model. Given latent class *g*, the survival submodel is specified as

(3) $$\begin{array}{c} h_{i}\left(\;t\;|\;c_{i}=g\;\right)=h_{0g}\left(\;t\;\right)\exp\left(\;X_{ei}\left(\;t\;\right)\delta_{g}+\gamma_{g}Z_{ig}^{*}\left(\;t_{ij}\;\right)\;\right), \end{array}$$

where *h*_0*g*_(*t*) is the baseline hazard function for class *g* and *X*_*ei*_(*t*) is the covariate vector associated with the *r*-vector parameters *δ*_*g*_ for the latent class *g*.

The quantity, *Z*_*ig*_^*∗*^(*t*_*ij*_) = *Z*_*i*_(*t*_*ij*_)*b*_*ig*_ + *X*_*i*_(*t*_*ij*_)*β*_*g*_, is the trajectory of the longitudinal function for class *g* to connect the longitudinal process with the survival outcome. The parameter *γ*_*g*_ links the longitudinal and time-to-event outcomes in each class.

### 2.2. Simulation Studies

We conducted this simulation study to examine bias, SE, the average bias of parameters estimation (AB-PE), the average SE of parameters estimation (ASE-PE), and coverage probability of the 95% confidence interval (CP) for three approaches (PA, JLCM, and SA) for the longitudinal and survival submodels. AB-PE shows the average of absolute bias of all parameters estimation. CP shows the proportion of time that confidence interval contains the true value.

A multinomial logistic model was considered for the latent class membership for each subject:

(4) $$\begin{array}{c} \pi_{ig}=P\left(\;c_{i}=g\;|\;x_{1},x_{2}\;\right)=\frac{\exp\left(-0.5+x_{1}+x_{2}\right)}{\sum_{l=1}^{2}\exp\left(-0.5+x_{1}+x_{2}\right)}. \end{array}$$

We considered a binary and a continuous covariate, where *x*_1_ is called a treatment effect, which was assumed as a binomial distribution with *P* = 0.5 and *x*_2_ ~ *N*(0,1). We assumed two latent classes (*g* = 2), where approximately 50% of the subjects belonged to class 1.

The longitudinal outcome was generated from a linear mixed model, where time of measurements was fixed at *t* = 0,0.5,1, 1.5,2, 2.5,3,…, 5 with a maximum of 11 measurements. The longitudinal submodel given to each latent class is

(5) Yitijci=g=β0g+β1gtij+β2gx1+β3gx2+big+εij,g=Zig∗+εij,g.

To achieve appropriate heterogeneous classes and to decrease misclassification rate, we considered the parameters with opposite direction in two classes from a previous study [13]. Thus, in the first class, we set coefficients to be (*β*_01_, *β*_11_, *β*_21_, *β*_31_) = (1, −1,0.5,1) and assumed subject-specific unobservable heterogeneity in class 1, *b*_*i*1_ ~ *N*(0,1). The error term had normal standard distribution. In the second class, we set coefficients to be (*β*_02_, *β*_12_, *β*_22_, *β*_32_) = (−1,1, −0.5, −1) and assumed that *b*_*i*2_ ~ *N*(0,1), and *ε*_*ij*,2_ ~ *N*(0,0.5) where the random intercept effect was assumed independent from the error term. The *Z*_*ig*_^*∗*^ = *β*_0*g*_ + *β*_1*g*_*t*_*ij*_ + *β*_2*g*_*x*_1_ + *β*_3*g*_*x*_2_ + *b*_*ig*_ is called trajectory function for each class.

The survival submodel assumed a Cox model with a Weibull baseline hazard function. The event time was generated using an inverse cumulative hazard function [15, 28, 29]. The censored time is noninformative and is uniformly distributed random variable on 2.5+ uniform [0,3]. Therefore, the observed failure time for the *i*th subject was considered as the minimum of true event time and censored time [20, 30]. As some previous studies, the censoring rate was considered around 60% in this simulation study [13, 28].

The survival submodel was generated for each latent class as follows:

(6) $$\begin{array}{c} h_{i}\left(\;t\;|\;c_{i}=g\;\right)=\alpha_{g}\lambda_{g}t^{\alpha_{g}-1}\exp\left(\;\delta_{g}x_{1}+\gamma Z_{ig}^{*}\;\right). \end{array}$$

The treatment effect on the time-to-event was *δ* = 0.5 and −0.5 in classes 1 and 2, respectively. The shape and scale parameters, (*α*, *λ*), of baseline hazard function were (0.6, 0.001) and (1, 0.001) in classes 1 and 2, respectively.

Sets of simulated data were performed for three sample sizes (150, 300, and 600 as small, moderate, and large sample sizes). Similar to previous study, three association parameters between longitudinal and survival outcomes were considered *γ* = (0,0.2,0.5) for none, moderate, and considerable association, respectively [12]. The magnitude of the association parameters was assumed the same in the two classes. For each simulation, the three approaches of PA, SA, and JLCM were fitted. We ran 1000 replications for each set of simulated data.

There are several methods to estimate parameters in joint models, including ML, restricted maximum likelihood (REML), and Bayesian method [18]. In PA, Gauss-Hermite integration method for maximizing the log likelihood of the joint distribution and EM iterations algorithm or quasi-Newton iterations were used. In JLCM, ML with EM algorithm was implemented to estimate parameters. For SA approach, ML in the longitudinal submodel and REML in the survival submodel were used for parameters estimation. The JM and LCMM packages in R version 3.1.1 software were used in this study.

## 3. Results of Simulation Study

### 3.1. Effect of Sample Size

**Figure 1:**
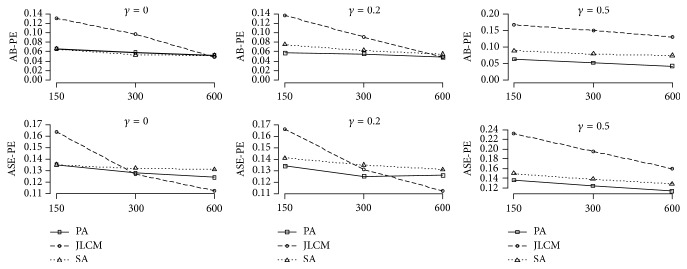
Comparisons of AB-PE and ASE-PE for the longitudinal submodel for the three approaches with different sample sizes (150, 300, and 600) and different association parameters (0, 0.2, and 0.5). PA: proposed approach; JLCM: joint latent class model; SA: separate approach.

**Figure 2:**
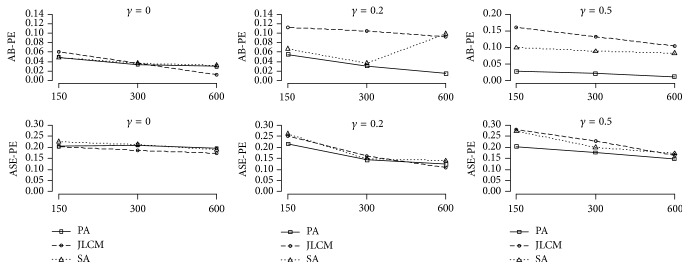
Comparisons of AB-PE and ASE-PE for the treatment effect on the survival submodel for the three approaches with different sample sizes (150, 300, and 600) and different association parameters (0, 0.2, and 0.5). PA: proposed approach; JLCM: joint latent class model; SA: separate approach.

Simulations results showed that in most cases when the three approaches were used the sample size increased, while AB-PE and ASE-PE decreased, and the CP went close to nominal level of 0.95. Tables 1–3 and Figures 1 and 2 present detailed information.

### 3.2. Effect of Association between the Longitudinal and Survival Outcomes

#### 3.2.1. None-Association (*γ* = 0)

For JLCM, the model with the best BIC and the CI assumption satisfied included two latent classes (*g* = 2) for the moderate and large sample sizes, while in the small sample size, *g* = 1 was the best-fit model. For the small sample size, results were reported for the two classes, since we can compare the models together. The average misclassification rates for the two latent classes for sample sizes of 150, 300, and 600 were 24%, 5%, and 1.4%, respectively.

AB-PE and ASE-PE for the longitudinal submodel of PA and SA for the small sample size were the same and the lowest among the three approaches. Additionally, PA had lower ASE-PE than SA for the moderate and large sample sizes. For the large sample size, AB-PE of the longitudinal submodel for the three approaches was the same, and JLCM had the lowest ASE-PE (Figure 1).

AB-PE for the treatment effect on time-to-event in the PA and SA was approximately the same for the small and moderate sample sizes. In addition, PA had better CP as compared with SA. Besides, for the large sample size, AB-PE and ASE-PE for the treatment effect on the survival submodel of JLCM were the lowest (Figure 2) and had a good CP amongst the three approaches.

The average of absolute bias and SE for the association parameter of PA and SA was approximately the same, and by increasing the sample size, bias and its SE decreased.

Bias, SE, and CP estimated parameters for the three approaches are presented in Table 1. AB-PE and their ASE-PE for the longitudinal and survival submodels are shown in Figures 1 and 2.

#### 3.2.2. Moderate Association (*γ* = 0.2)

For JLCM, the average misclassification rate for the two latent classes in the small sample size was approximately 20%, which was greater than the other sample sizes.

PA had the smallest AB-PE and ASE-PE for the longitudinal submodel among the three approaches for small and moderate sample sizes. As for the large sample size, PA and JLCM had the same AB-PE, but JLCM had smaller ASE-PE in comparison with the other approaches for the longitudinal submodel (Figure 1).

AB-PE for the treatment effect on the survival submodel of PA was lower than JLCM and SA for the all sample sizes. In addition, ASE-PE of PA was the lowest among the three approaches for the small and moderate sample sizes. Furthermore, JLCM had the lowest ASE-PE, and SA had the highest AB-PE among the three approaches for the large sample size (Figure 2).

Figures 1 and 2 show the results. In addition, by increasing the sample size, CP for PA and JLCM were close to 0.95 (Table 2).

#### 3.2.3. Considerable Association (*γ* = 0.5)

JLCM with one to three numbers of latent classes was performed. For the moderate and large sample sizes, the three appropriate numbers of latent classes were detected based on the best BIC, and satisfaction CI assumption, and for the small sample size, one latent class was preferred. We reported the estimation of parameters for the two classes in order to compare the three approaches together. The average misclassification rates for the two latent classes were 47%, 26%, and 10%, for the small, moderate, and large sample sizes, respectively.

PA had the lowest AB-PE and ASE-PE and plausible CP for the longitudinal outcome, as well as the treatment effect on the survival submodel for the three sample sizes. In the three approaches, if sample size increases, AB-PE and ASE-PE decrease (Figures 1 and 2) and the CP get closer to nominal level of 0.95. In addition, bias of association parameter for PA and SA was negative in two classes. Moreover, the average absolute bias of association parameter for SA was higher than PA. The average of CP for PA, JLCM, and SA was 0.970, 0.837, and 0.833, for the large sample size, respectively. For bias, SE, and CP information of parameters estimation, refer to Table 3.

## 4. Empirical Example

### 4.1. The Data and Methods Description

The number of new HIV infections has declined by 38% worldwide from 2001 to 2013, followed by a significant decline in AIDS-related deaths [31]. According to the World Health Organization [32] report, 36.7 million people will be living with HIV/AIDS by the end of 2015 [32].

Among infectious diseases, the HIV/AIDS studies are a good example to be used in joint modeling of the longitudinal and survival processes. There are some literatures available that have used the joint modeling on such data [3, 6, 33, 34]. In HIV/AIDS studies, CD4 cells are considered as a sign of disease progression in HIV-infected people. CD4 cells help to coordinate the immune system's response to certain microorganisms such as viruses; a low CD4 count is an indication of a higher risk of infection [6, 33, 35].

In this study, the HIV/AIDS dataset from Community Programs for Clinical Research on AIDS (CPCRA) was used [36], and a total of 467 patients infected with HIV were included in this study. The two outcomes were the longitudinal measurements of CD4, recorded at different time points: at the study entry, 2, 6, 12, and 18 months, and the time-to-death outcome. In CPCRA study, patients received two treatments, Zalcitabine (ddC) or Didanosine (ddI), randomly. Only a brief description of the dataset used in this study was mentioned here, since they have been fully described elsewhere [36].

In the present study, the HIV/AIDS dataset was used as an example to evaluate PA. To predict the class membership, an intercept-only-model or different covariates such as baseline hemoglobin (Hgb), treatment, and gender were considered from the literature [6, 13, 18]. In this study, based on Hgb and the treatment covariates, the class membership probability for each patient was identified via latent class framework. Then the patients were divided into two latent classes based on their highest posterior class membership probabilities. The number of latent classes was chosen in a way that there were enough observations in each latent class for easier classification, consistency with our simulation-based study, and easier interpretation.

PA and SA for modeling the influence of effective covariates on CD4 count and time-to-death were conducted in each class. In addition, we fitted JLCM for longitudinal CD4 measures and time-to-death with the number of latent classes varying from 1 to 3.

Due to the skewed distribution of CD4 cell level, and the presence of zero values, log(CD4+1) was used as the longitudinal outcome. The baseline hazard functions were estimated by Weibull distribution.

We used latent GOLD software ver. 4.5 to identify the probability of the class membership for each subject and influential covariates on classes.

### 4.2. Results of Application Data

The results of latent class framework, using the PA and SA, showed that Hgb was significant (*p* value < 0.001), while the treatment (*p* value = 0.170) was an insignificant covariate on the subtype classification. Based on the classification, 51% of the patients were in the first class.

*The PA on HIV/AIDS Dataset*. In both classes, CD4 values decreased with time. The estimates of association parameters between CD4 and the time-to-death (*γ*) were significant and negative in both classes. Treatment had a significant effect on time-to-death in the second class. The effective covariates on the longitudinal and survival submodels are presented in Table 4.

**Figure 3:**
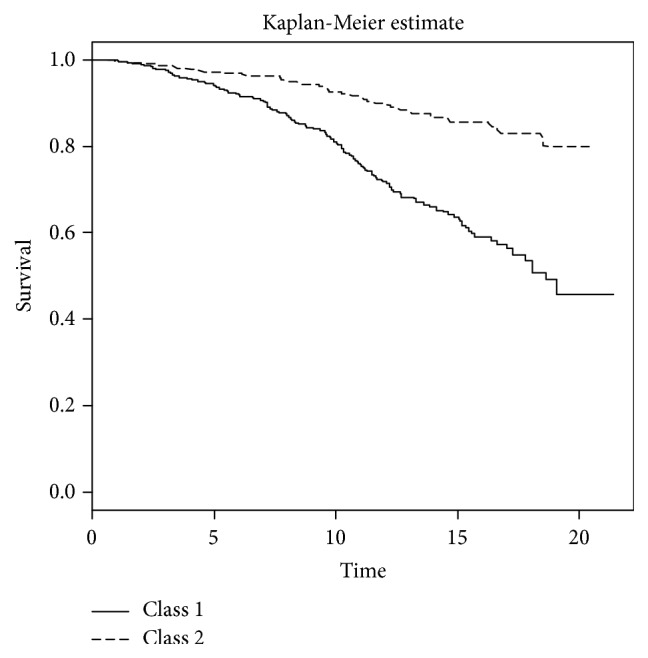
Kaplan-Meier survival plot for the two classes on the HIV/AIDS patients.

**Figure 4:**
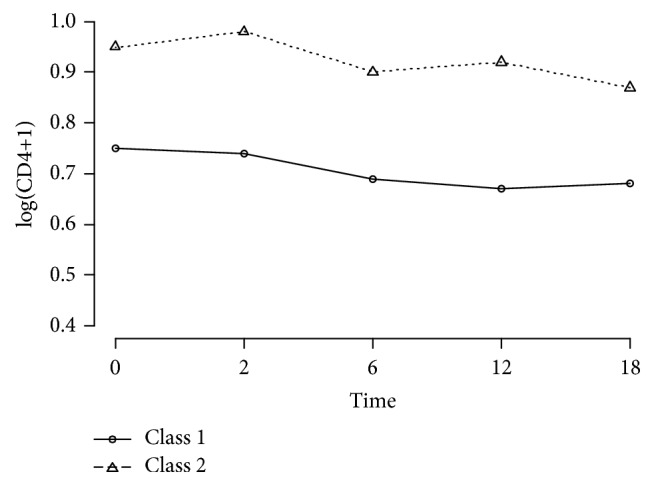
Mean log(CD4+1) over time for the two latent classes on the HIV/AIDS patients.

The Kaplan-Meier survival plot and the mean of log(CD4+1) stratified by posterior classification are presented in Figures 3 and 4. Patients in the second class had a better survival rate and higher log(CD4+1) values. 

*The SA on HIV/AIDS Dataset*. In the longitudinal submodel, time effect in the first class occurrence reduces CD4 cells significantly. Hgb had a small, but significant effect on CD4 in the first class and a strong significant effect in the second class. In the survival submodel, the treatment had a significant effect on time-to-death in the second class. The estimated association parameters between CD4 and the time-to-death were significant and negative in both classes. The results for SA are presented in Table 4. 

*The JLCM on HIV/AIDS Dataset*. BIC calculated for two latent classes was 4280.560, which was smaller than one class (4417.210) and three classes (4304.480). Also, the CI assumption was not rejected (*p* value = 0.250) for this model; thus, the model with two latent classes was preferred. The probability of belonging to the latent classes was not significantly associated with the treatment (*p* value = 0.370) and Hgb (*p* value = 0.566).

In the longitudinal submodel, the time effect was negative and significant in the two latent classes. In the survival submodel, the treatment in the second class and Hgb in the first class were significantly associated with risk of death for HIV/AIDS patients (Table 4).

Overall, ASE-PE for the longitudinal submodel was 0.024, 0.024, and 0.039 for the PA, SA, and JLCM, respectively. Furthermore, ASE-PE for the survival submodel among the three approaches were 0.106, 0.394, and 0.343 for the PA, SA, and JLCM, respectively.

## 5. Discussion

### 5.1. Discussions about Simulation Results

According to the simulations results, in most cases, in the three approaches when the sample size increased, AB-PE and ASE-PE decreased, and CP got closer to nominal level of 0.95. This finding is consistent with a simulation-based study for a parametric latent class joint model of the longitudinal and survival outcome [2].

Our main finding occurred when there was a considerable association (*γ* = 0.5) between two processes. PA provided lower AB-PE and ASE-PE than JLCM and SA for the three sample sizes; hence, PA yielded unbiased and more efficient estimation of parameters than JLCM and SA for the longitudinal and survival submodels. The results of a similar study are consistent with those of PA for heterogeneous populations [13]. However, PA used the full longitudinal trajectory to connect the longitudinal and survival data, whereas in the similar study, only the shared random effect was used. This study showed that the model worked well in estimating longitudinal and survival parameters in a sample size of 400 and for the considerable association between the two processes.

To the best of our knowledge, no comparison has been made between JLCM and other approaches for the heterogeneous populations. However, to compare with similar studies, we used the ones that had assumed that the subjects exhibited one pattern. For comparison between PA and SA, the results are consistent with previous studies that had conducted simulation-based studies where there was a strong association between the two outcomes. Their results showed that the joint modeling that utilizes information from both outcomes tends to produce almost unbiased estimates and smaller SEs of all the parameters than separate model [8, 37, 38]. Furthermore, since AB-PE and ASE-PE in JLCM were higher than PA, it seems that JLCM cannot contain the strength of association entirely by latent structures. In addition, the number of latent classes in JLCM could not be estimated directly and for some sample sizes, the appropriate number of classes is selected according to lower BIC, and acceptance of the CI assumption was not consistent with the true size of classes. Therefore, it led to biased estimation of parameters, while PA achieved an appropriate number of latent classes directly with no need for the CI assumption and BIC criterion. In addition, the association parameter for SA was underestimated in comparison with PA. This result concurs with those of a similar study which showed that using the longitudinal outcome as a time-varying covariate into the survival model is not recommended, due to severe underestimation of the association parameter [15].

When there was the moderate correlation (*γ* = 0.2) between the longitudinal and survival processes, PA was preferred over JLCM and SA for the small and moderate sample sizes. In addition, the average misclassification rate for the small sample size in JLCM was high; hence, AB-PE and ASE-PE were increased. Furthermore, for the large sample size, the average misclassification rate for JLCM was low; thus, AB-PE of the longitudinal submodel for JLCM and PA was the same and JLCM was more efficient.

For the case of none-association (*γ* = 0) between the longitudinal and survival processes, results of the longitudinal submodel of PA were similar to SA in the small sample size. Our finding is consistent with a similar study for none-association between the longitudinal and survival data [39]. Also, PA was more efficient than SA in the moderate and large sample sizes. For the small and moderate sample sizes, the results of the effect of the treatment on the time-to-event of PA and SA were found to be similar. This result is consistent with similar studies that had shown when there was no association between the longitudinal and survival data; the longitudinal information did not improve the estimation of the treatment effect on the survival outcome [12, 39]. Moreover, JLCM was unbiased and more efficient than the other two approaches in the large sample size. Computationally, JLCM was faster and easier than the time consuming PA. In addition, for the large sample size, the misclassification rate was the lowest; hence, the entire association between the longitudinal and survival outcomes can be considered with the latent structure. Therefore, in this case, JLCM was more desirable than the two other approaches.

We believe that our PA can address the heterogeneity and consider the association structure behind the longitudinal and survival processes. One of the advantages of using PA was its capability to reduce AB-PE and ASE-PE by increasing the sample size and intensity of the association parameter. However, it leads to increased computation and time required to fit the model, which is one of the disadvantages of PA.

Finally, this study had some limitations that have to be addressed. First, in this study, we used the same magnitude parameters with opposite direction to consider the two heterogeneous classes. Second, association parameters for the two classes were the same. Third, this study was limited to two latent classes and continuous longitudinal and single event data. Further researches have to use PA with various options for the survival and longitudinal processes such as a nonlinear mixed model for the longitudinal data and a parametric or recurrent survival model. Moreover, we used ML estimation, while the Bayesian inference can be an alternative approach for estimation of parameters. Also, further simulation studies can be performed to evaluate statistical properties for PA including the statistical power.

### 5.2. Discussions about HIV/AIDS Results

The results showed that Hgb was a significant covariate in classifying subjects via latent class framework, concurring with the results of a study on this dataset [13].

According to the results of the application, the time effect was significant in each class for CD4 longitudinal outcome in PA and JLCM. This study produced results which corroborate the findings of a great deal of the previous works that used this dataset [3, 13]. There were no statistically significant differences between the two treatments (ddC and ddI), on CD4 longitudinal outcome in the two latent classes in the three approaches. This result agrees with the findings of similar studies that had investigated the effect of the treatment on CD4 longitudinal outcome [11, 13]. In addition, Hgb had a significant positive effect on CD4 values in both classes in PA and SA. This result matches those observed in earlier studies on this dataset [13].

As for the survival submodel, the treatment was a significant factor on time-to-death in the second class in the three approaches. Patients in the second class had a better survival rate when given ddC. Furthermore, Hgb was not a significant factor of the death rate in the two classes for PA and SA. This finding is consistent with a similar study where Hgb was imported into the model [13]. In JLCM, Hgb was a significant factor of death rate in the first class but did not have a significant effect in the second class.

The estimated association parameters (*γ*_1_ and *γ*_2_) between CD4 and time-to-death were negative and significant in both classes for PA and SA. This implies that a higher CD4 count is associated with a lower death rate or a reduced number of CD4 significantly increases the risk of death in patients [10, 40].

Overall, the results of PA in this study confirm those of the previous studies on this dataset and with the biomedical literature [11, 13, 14, 18]. Moreover, PA and SA had the same ASE-PE approximately for the longitudinal submodel that are consequently more efficient than JLCM. In addition, PA had lower ASE-PE for the survival submodel; hence, PA is more efficient than the other two approaches for indicating the influence covariates on time-to-death in patients with HIV/AIDS. The results of the three approaches on CPCRA data confirm our result in the simulation study when there was a considerable association parameter between the longitudinal outcome and time-to-event in the large sample size.

The application study on CPCRA data shows the advantages of our PA. Therefore, by using appropriate latent class joint model, we can assign treatment ddC to patients with a higher chance of being classified into the second class based on their baseline hemoglobin (Hgb), thereby increasing the survival rate. In other cases, when the treatments have side effects, we could utilize an appropriate latent class joint modeling to identify a subgroup of patients that are most likely to have side effects. Hence, we can assign treatments in a personalized manner to avoid such subgroup, which can further benefit the patients.

## 6. Conclusion

This simulation-based study provided an approach for the joint model, by considering the association between the longitudinal and time-to-event data for heterogeneous populations which does not require the CI assumption. This study concluded that for the three approaches when the sample size increased, AB-PE and ASE-PE decreased to some extent, and CP reached the nominal level of 0.95. Finally, when there were a considerable association and the large sample size, PA was preferred.

## Figures and Tables

**Table 1:** Comparison of the estimation of parameters in the three approaches in different sample sizes, *γ* = 0.

Models	Class	Parameter	True value	*N* = 150			*N* = 300			*N* = 600		
				Bias	SE	CP	Bias	SE	CP	Bias	SE	CP
JLCM	Class 1	Intercept	1	0.118	0.178	0.932	0.099	0.143	0.941	0.033	0.121	0.953
		*t*_*ij*_	−1	0.111	0.294	0.923	0.086	0.208	0.934	0.041	0.197	0.963
		*X*_1_	0.5	−0.178	0.143	0.942	−0.101	0.122	0.944	−0.065	0.119	0.959
		*X*_2_	1	−0.121	0.138	0.921	−0.098	0.120	0.949	−0.045	0.119	0.971
		var⁡(*b*_1*i*_)	1	0.128	0.044	0.912	0.104	0.022	0.952	0.059	0.012	0.967
		*X*_1_	*0.5*	*0.068*	*0.210*	*0.931*	*0.042*	*0.183*	*0.944*	*0.013*	*0.174*	*0.953*
	Class 2	Intercept	−1	0.119	0.184	0.918	0.089	0.147	0.919	0.031	0.129	0.955
		*t*_*ij*_	1	0.112	0.329	0.921	0.097	0.226	0.941	0.042	0.184	0.961
		*X*_1_	−0.5	0.182	0.153	0.932	0.112	0.141	0.948	0.061	0.108	0.957
		*X*_2_	−1	0.123	0.139	0.927	0.079	0.124	0.954	0.048	0.124	0.974
		var⁡(*b*_1*i*_)	1	0.124	0.038	0.922	0.112	0.018	0.922	0.067	0.009	0.962
		*X*_1_	*−0.5*	*0.053*	*0.199*	*0.929*	*0.031*	*0.187*	*0.929*	*0.011*	*0.176*	*0.958*

PA	Class 1	Intercept	1	0.058	0.164	0.937	0.049	0.151	0.951	0.037	0.149	0.953
		*t*_*ij*_	−1	0.059	0.214	0.925	0.051	0.205	0.953	0.049	0.198	0.959
		*X*_1_	0.5	−0.079	0.130	0.943	−0.071	0.124	0.961	−0.069	0.120	0.959
		*X*_2_	1	−0.082	0.127	0.920	−0.074	0.118	0.968	−0.052	0.118	0.970
		var⁡(*b*_1*i*_)	1	−0.061	0.023	0.918	−0.061	0.016	0.954	−0.059	0.015	0.956
		*X*_1_	*0.5*	*0.062*	*0.198*	*0.929*	*0.040*	*0.210*	*0.959*	*0.033*	*0.197*	*0.951*
		*γ*	*0*	*0.032*	*0.197*	*0.931*	*0.001*	*0.187*	*0.951*	*0.000*	*0.178*	*0.953*
	Class 2	Intercept	−1	0.068	0.159	0.932	0.042	0.151	0.962	0.032	0.150	0.954
		*t*_*ij*_	1	0.051	0.228	0.926	0.049	0.226	0.956	0.047	0.220	0.958
		*X*_1_	−0.5	0.073	0.147	0.941	0.061	0.142	0.961	0.057	0.131	0.954
		*X*_2_	−1	0.057	0.129	0.918	0.054	0.127	0.978	0.050	0.123	0.971
		var⁡(*b*_1*i*_)	1	0.073	0.029	0.924	0.072	0.018	0.954	0.069	0.015	0.955
		*X*_1_	*−0.5*	*0.037*	*0.212*	*0.927*	*0.029*	*0.211*	*0.951*	*0.027*	*0.196*	*0.952*
		*γ*	*0*	*0.021*	*0.194*	*0.927*	*0.000*	*0.191*	*0.949*	*0.000*	*0.167*	*0.955*

SA	Class 1	Intercept	1	0.058	0.164	0.912	0.049	0.158	0.911	0.038	0.157	0.951
		*t*_*ij*_	−1	0.060	0.212	0.823	0.052	0.210	0.852	0.050	0.210	0.899
		*X*_1_	0.5	−0.079	0.131	0.910	−0.073	0.127	0.924	−0.068	0.126	0.949
		*X*_2_	1	−0.083	0.124	0.847	−0.074	0.121	0.868	−0.053	0.119	0.890
		var⁡(*b*_1*i*_)	1	−0.062	0.024	0.836	−0.060	0.022	0.854	−0.057	0.021	0.860
		*X*_1_	*0.5*	*0.061*	*0.224*	*0.798*	*0.043*	*0.214*	*0.859*	*0.035*	*0.194*	*0.878*
		*γ*	*0*	*0.032*	*0.281*	*0.842*	*0.001*	*0.198*	*0.851*	*0.000*	*0.163*	*0.880*
	Class 2	Intercept	−1	0.068	0.158	0.910	0.044	0.156	0.912	0.032	0.156	0.932
		*t*_*ij*_	1	0.052	0.236	0.814	0.049	0.229	0.825	0.046	0.232	0.910
		*X*_1_	−0.5	−0.073	0.149	0.894	0.063	0.146	0.861	0.056	0.143	0.951
		*X*_2_	−1	0.058	0.127	0.818	0.052	0.126	0.833	0.051	0.123	0.949
		var⁡(*b*_1*i*_)	1	0.072	0.026	0.911	0.071	0.020	0.909	0.068	0.021	0.954
		*X*_1_	*−0.5*	*0.037*	*0.228*	*0.782*	*0.031*	*0.213*	*0.810*	*0.029*	*0.187*	*0.894*
		*γ*	*0*	*−0.021*	*0.273*	*0.846*	*−0.000*	*0.179*	*0.829*	*−0.000*	*0.157*	*0.878*

JLCM: joint latent class models; PA: proposed approach; SA: separate approach; SE: standard error; CP: coverage probability of the 95% confidence interval.

**Table 2:** Comparison of the estimation of parameters in the three approaches for different sample sizes, *γ* = 0.2.

Models	Class	Parameter	True value	*N* = 150			*N* = 300			*N* = 600		
				Bias	SE	CP	Bias	SE	CP	Bias	SE	CP
JLCM	Class 1	Intercept	1	0.128	0.189	0.949	0.087	0.149	0.948	0.033	0.137	0.967
		*t*_*ij*_	−1	0.119	0.297	0.937	0.079	0.201	0.958	0.042	0.184	0.974
		*X*_1_	0.5	−0.183	0.148	0.934	−0.101	0.126	0.953	−0.064	0.117	0.953
		*X*_2_	1	−0.134	0.137	0.948	−0.097	0.117	0.951	−0.051	0.110	0.952
		var⁡(*b*_1*i*_)	1	0.129	0.046	0.946	0.077	0.024	0.949	−0.037	0.009	0.951
		*X*_1_	*0.5*	*0.123*	*0.189*	*0.935*	*0.111*	*0.171*	*0.957*	*0.099*	*0.106*	*0.917*
	Class 2	Intercept	−1	0.129	0.176	0.941	0.091	0.152	0.925	0.035	0.129	0.953
		*t*_*ij*_	1	0.116	0.317	0.934	0.086	0.215	0.954	0.046	0.199	0.966
		*X*_1_	−0.5	0.178	0.158	0.929	0.107	0.141	0.955	0.051	0.122	0.953
		*X*_2_	−1	0.127	0.142	0.911	0.083	0.122	0.951	0.053	0.109	0.977
		var⁡(*b*_1*i*_)	1	0.126	0.051	0.923	0.099	0.019	0.937	0.067	0.007	0.961
		*X*_1_	*−0.5*	*0.104*	*0.311*	*0.928*	*0.098*	*0.149*	*0.942*	*0.086*	*0.115*	*0.973*

PA	Class 1	Intercept	1	0.047	0.165	0.931	0.041	0.148	0.948	0.033	0.141	0.957
		*t*_*ij*_	−1	0.051	0.213	0.919	0.049	0.199	0.928	0.043	0.197	0.954
		*X*_1_	0.5	−0.063	0.131	0.934	−0.063	0.123	0.958	−0.064	0.120	0.961
		*X*_2_	1	−0.072	0.127	0.949	−0.066	0.118	0.961	−0.051	0.118	0.973
		var⁡(*b*_1*i*_)	1	−0.059	0.023	0.950	−0.056	0.016	0.946	−0.037	0.014	0.977
		*X*_1_	*0.5*	*0.063*	*0.219*	*0.949*	*0.031*	*0.148*	*0.943*	*0.013*	*0.121*	*0.969*
		*γ*	*0.2*	*−0.022*	*0.204*	*0.951*	*−0.018*	*0.119*	*0.950*	*−0.001*	*0.101*	*0.952*
	Class 2	Intercept	−1	0.048	0.158	0.938	0.045	0.150	0.949	0.032	0.147	0.953
		*t*_*ij*_	1	0.049	0.228	0.944	0.046	0.221	0.952	0.046	0.216	0.954
		*X*_1_	−0.5	0.066	0.147	0.939	0.062	0.138	0.964	0.051	0.131	0.959
		*X*_2_	−1	0.056	0.128	0.938	0.054	0.120	0.975	0.054	0.120	0.981
		var⁡(*b*_1*i*_)	1	0.061	0.023	0.921	0.068	0.017	0.951	0.069	0.017	0.972
		*X*_1_	*−0.5*	*0.047*	*0.214*	*0.933*	*0.031*	*0.138*	*0.952*	*0.017*	*0.127*	*0.988*
		*γ*	*0.2*	*0.024*	*0.118*	*0.918*	*0.011*	*0.121*	*0.954*	*−0.001*	*0.111*	*0.979*

SA	Class 1	Intercept	1	0.063	0.167	0.901	0.049	0.157	0.939	0.038	0.158	0.941
		*t*_*ij*_	−1	0.067	0.213	0.849	0.052	0.216	0.882	0.050	0.210	0.901
		*X*_1_	0.5	−0.084	0.141	0.921	−0.073	0.129	0.849	−0.069	0.127	0.924
		*X*_2_	1	−0.095	0.129	0.899	−0.074	0.124	0.878	−0.053	0.117	0.901
		var⁡(*b*_1*i*_)	1	−0.079	0.025	0.848	−0.060	0.027	0.851	−0.057	0.022	0.823
		*X*_1_	*0.5*	*0.071*	*0.278*	*0.826*	*0.043*	*0.158*	*0.831*	*0.101*	*0.143*	*0.689*
		*γ*	*0.2*	*−0.151*	*0.354*	*0.911*	*−0.121*	*0.131*	*0.829*	*−0.103*	*0.112*	*0.634*
	Class 2	Intercept	−1	0.089	0.171	0.914	0.068	0.158	0.899	0.042	0.157	0.864
		*t*_*ij*_	1	0.066	0.241	0.862	0.061	0.228	0.841	0.058	0.231	0.899
		*X*_1_	−0.5	0.074	0.157	0.863	0.069	0.164	0.866	0.061	0.144	0.942
		*X*_2_	−1	0.061	0.134	0.848	0.056	0.127	0.839	0.054	0.125	0.956
		var⁡(*b*_1*i*_)	1	0.076	0.035	0.926	0.071	0.023	0.914	0.069	0.020	0.873
		*X*_1_	*−0.5*	*0.064*	*0.246*	*0.816*	*0.031*	*0.141*	*0.821*	*0.097*	*0.138*	*0.614*
		*γ*	*0.2*	*−0.141*	*0.297*	*0.721*	*−0.119*	*0.117*	*0.832*	*−0.097*	*0.114*	*0.633*

JLCM: joint latent class models; PA: proposed approach; SA: separate approach; SE: standard error; CP: coverage probability of the 95% confidence interval.

**Table 3:** Comparison of the estimation of parameters in the three approaches for different sample sizes, *γ* = 0.5.

Models	Class	Parameter	True value	*N* = 150			*N* = 300			*N* = 600		
				Bias	SE	CP	Bias	SE	CP	Bias	SE	CP
JLCM	Class 1	Intercept	1	0.131	0.254	0.834	0.129	0.244	0.845	0.112	0.237	0.887
		*t*_*ij*_	−1	0.141	0.311	0.832	0.134	0.221	0.856	0.114	0.189	0.904
		*X*_1_	0.5	−0.215	0.265	0.901	−0.197	0.226	0.933	−0.174	0.136	0.957
		*X*_2_	1	−0.179	0.207	0.749	−0.153	0.187	0.770	−0.128	0.150	0.781
		var⁡(*b*_1*i*_)	1	0.164	0.119	0.756	0.141	0.101	0.762	−0.119	0.089	0.798
		*X*_1_	*0.5*	*0.144*	*0.231*	*0.631*	*0.122*	*0.210*	*0.629*	*0.103*	*0.159*	*0.711*
	Class 2	Intercept	−1	0.138	0.244	0.851	0.126	0.178	0.862	0.118	0.169	0.873
		*t*_*ij*_	1	0.152	0.331	0.766	0.148	0.245	0.884	0.128	0.219	0.905
		*X*_1_	−0.5	0.226	0.258	0.706	0.195	0.249	0.810	0.176	0.188	0.913
		*X*_2_	−1	0.168	0.198	0.721	0.142	0.188	0.784	0.117	0.123	0.823
		var⁡(*b*_1*i*_)	1	0.156	0.131	0.633	0.125	0.119	0.660	0.112	0.096	0.703
		*X*_1_	*−0.5*	*0.176*	*0.332*	*0.678*	*0.142*	*0.249*	*0.673*	*0.108*	*0.167*	*0.785*

PA	Class 1	Intercept	1	0.049	0.163	0.930	0.037	0.158	0.951	0.024	0.147	0.959
		*t*_*ij*_	−1	0.054	0.223	0.923	0.030	0.195	0.934	0.031	0.188	0.957
		*X*_1_	0.5	−0.072	0.137	0.938	−0.067	0.124	0.949	−0.051	0.120	0.969
		*X*_2_	1	−0.081	0.129	0.952	−0.069	0.105	0.947	−0.050	0.084	0.975
		var⁡(*b*_1*i*_)	1	−0.052	0.034	0.956	−0.046	0.025	0.954	−0.038	0.012	0.982
		*X*_1_	*0.5*	*0.024*	*0.208*	*0.941*	*0.019*	*0.171*	*0.947*	*0.012*	*0.142*	*0.976*
		*γ*	*0.5*	*−0.017*	*0.177*	*0.950*	*−0.012*	*0.153*	*0.951*	*−0.002*	*0.149*	*0.952*
	Class 2	Intercept	−1	0.043	0.157	0.927	0.039	0.154	0.951	0.026	0.149	0.955
		*t*_*ij*_	1	0.051	0.218	0.944	0.044	0.207	0.947	0.037	0.179	0.956
		*X*_1_	−0.5	0.068	0.153	0.935	0.057	0.133	0.960	0.048	0.124	0.962
		*X*_2_	−1	0.077	0.121	0.920	0.064	0.119	0.971	0.051	0.111	0.989
		var⁡(*b*_1*i*_)	1	0.085	0.029	0.931	0.067	0.022	0.962	0.063	0.018	0.973
		*X*_1_	*−0.5*	*0.032*	*0.201*	*0.933*	*0.024*	*0.183*	*0.949*	*0.013*	*0.155*	*0.982*
		*γ*	*0.5*	*−0.019*	*0.148*	*0.915*	*−0.015*	*0.137*	*0.951*	*−0.003*	*0.121*	*0.987*

SA	Class 1	Intercept	1	0.076	0.167	0.904	0.066	0.159	0.914	0.056	0.151	0.920
		*t*_*ij*_	−1	0.099	0.225	0.788	0.084	0.210	0.782	0.075	0.198	0.818
		*X*_1_	0.5	−0.084	0.153	0.901	−0.067	0.138	0.911	−0.059	0.129	0.915
		*X*_2_	1	−0.110	0.144	0.886	−0.108	0.114	0.878	−0.095	0.111	0.881
		var⁡(*b*_1*i*_)	1	−0.086	0.037	0.897	−0.077	0.031	0.858	−0.069	0.029	0.825
		*X*_1_	*0.5*	*0.101*	*0.288*	*0.728*	*0.091*	*0.179*	*0.813*	*0.084*	*0.157*	*0.817*
		*γ*	*0.5*	*−0.219*	*0.321*	*0.669*	*−0.210*	*0.276*	*0.671*	*−0.195*	*0.225*	*0.698*
	Class 2	Intercept	−1	0.066	0.169	0.907	0.059	0.161	0.895	0.058	0.158	0.877
		*t*_*ij*_	1	0.087	0.243	0.855	0.074	0.229	0.859	0.071	0.217	0.863
		*X*_1_	−0.5	0.074	0.172	0.873	0.065	0.168	0.899	0.063	0.148	0.912
		*X*_2_	−1	0.115	0.139	0.783	0.110	0.125	0.844	0.109	0.108	0.875
		var⁡(*b*_1*i*_)	1	0.091	0.055	0.845	0.089	0.041	0.873	0.082	0.035	0.863
		*X*_1_	*−0.5*	*0.098*	*0.259*	*0.709*	*0.086*	*0.219*	*0.691*	*0.082*	*0.189*	*0.693*
		*γ*	*0.5*	*−0.234*	*0.277*	*0.649*	*−0.210*	*0.228*	*0.718*	*−0.198*	*0.207*	*0.708*

JLCM: joint latent class models; PA: proposed approach; SA: separate approach; SE: standard error; CP: coverage probability of the 95% confidence interval.

**Table 4:** Comparison of the results for the three approaches on HIV/AIDS patients.

	Parameter name	PA			SA			JLCM		
		Estimates	SE	*p* value	Estimates	SE	*p* value	Estimates	SE	*p* value
Class 1	*Longitudinal submodel*									
	Intercept	0.626	0.054	<0.001	0.612	0.052	<0.001	1.057	0.134	<0.001
	Time	−0.011	0.002	<0.001	−0.006	0.002	0.002	−0.007	0.002	<0.001
	Treatment (ddC)	0.029	0.018	0.106	0.0282	0.018	0.124	−0.016	0.039	0.681
	Hgb	0.008	0.004	0.048	0.007	0.004	0.049	−0.001	0.007	0.815
	*Survival submodel*									
	Treatment (ddC)	0.055	0.120	0.646	0.151	0.170	0.373	−0.259	0.955	0.785
	Hgb	0.034	0.025	0.174	0.006	1.006	0.848	0.308	0.096	0.001
	*γ*_1_	−0.508	0.644	<0.001	−0.480	0.296	<0.001	-	-	-

Class 2	*Longitudinal submodel*									
	Intercept	1.163	0.086	<0.001	1.152	0.086	<0.001	0.708	0.098	<0.001
	Time	−0.006	0.002	0.003	−0.004	0.002	0.055	−0.011	0.002	<0.001
	Treatment (ddC)	0.021	0.022	0.345	0.022	0.022	0.329	0.004	0.028	0.877
	Hgb	−0.018	0.005	<0.001	−0.018	0.005	0.001	−0.002	0.006	0.709
	*Survival submodel*									
	Treatment (ddC)	−0.762	0.221	<0.001	−0.763	0.321	0.017	−0.558	0.263	0.034
	Hgb	0.059	0.059	0.317	0.031	0.082	0.702	−0.006	0.060	0.917
	*γ*_2_	−0.488	0.719	<0.001	−0.430	0.494	<0.001	-	-	-

JLCM: joint latent class models; PA: proposed approach; SA: separate approach; SE: standard error; Hgb: baseline hemoglobin values; ddC: Zalcitabine treatment.

## Abbreviations

JLCM: Joint latent class model
PA: Proposed approach
SA: Separate approach
BIC: Bayesian information criterion
CI: Conditional independence
CP: Coverage probability
SE: Standard error
AB-PE: Average bias of parameters estimation
ASE-PE: Average SE of parameters estimation
EM: Expected-Maximization
ML: Maximum likelihood
REML: Restricted maximum likelihood
CPCR: Community Programs for Clinical Research on AIDS
ddC: Zalcitabine
ddI: Didanosine.
